# Note from Dr. Howeth, President North Texas Medical Society

**Published:** 1890-12

**Authors:** V. A. Howeth

**Affiliations:** President N. T. Med. Association; Gainesville, Texas


GAINESVILLE, TEXAS, Nov. 17, 1890.

*Editor Daniel's Texas Medical Journal:*

On page 332 of the Transactions of the Texas State Medical Association, 1890, the Publishing Committee, by mistake, placed the name of B. R. Thomason, M. D., upon the list as an accredited delegate from the North Texas Medical Association. Will you publish this correction in the JOURNAL, as no delegates were appointed by said Association?

Yours truly,

V. A. HOWETH, M. D.,
President N. T. Med. Association.

[Referring to the above, it was perhaps a clerical error of the Judicial Council in reporting upon the accredited delegates. —ED.]

